# Comparison of LISA and INSURE techniques for surfactant administration: a multicentre retrospective study

**DOI:** 10.1007/s00431-026-07118-8

**Published:** 2026-06-03

**Authors:** Raffaella Panza, Rossella Caravita, Luigia Valenzano, Domenico Martinelli, Ilaria Farella, Pietro Guida, Michele Quercia, Giuseppe Latorre, Nicola Laforgia

**Affiliations:** 1 Neonatology and Neonatal Intensive Care Unit, Department of Interdisciplinary Medicine, “Aldo Moro” University of Bari, Bari, Italy; 2 Neonatal Intensive Care Unit, Department of Women’s and Children’s Health , Di Venere Hospital, Bari, Italy; 3 Neonatology and Neonatal Intensive Care Unit, Ecclesiastical General Hospital “F. Miulli”, Acquaviva Delle Fonti, Italy; 4 Cardiology Department, Ecclesiastical General Hospital “F. Miulli”, Acquaviva Delle Fonti, Italy

**Keywords:** INSURE, LISA, Surfactant, NIV

## Abstract

NIV and early targeted endotracheal surfactant administration play a pivotal role in the treatment of RDS and other respiratory conditions in neonates. LISA and INSURE are the most widely used techniques for surfactant delivery. This multicentre retrospective registry-based cohort study, conducted in three Southern Italy Neonatal Intensive Care Units between January 2024 and March 2025, aimed to compare their effectiveness. Neonates who received NIV soon after birth and rescue surfactant via either INSURE or LISA were included. Treatment failure was defined as the need for a second dose of surfactant or the need for intubation and mechanical ventilation within 72 h of life. The secondary outcomes were the incidence of BPD at 36 weeks post-menstrual age or at discharge and impact of the applied NIV mode on outcomes within the LISA subgroup. Sixty-one neonates were enrolled: 29 LISA group (48%) and 32 INSURE group (52%). No significant differences were observed in the need for a second surfactant dose (LISA 38% vs INSURE 19%, *p* = 0.095) or mechanical ventilation within the first 72 h of life (LISA 17% vs INSURE 9%, *p* = 0.460). BPD incidence was low and comparable between groups (LISA 7% vs INSURE 3%, *p* = 0.600). In LISA-treated neonates, HFNC, nCPAP, and NIPPV showed comparable effects on primary and secondary outcomes, while oxygen supplementation duration was significantly shorter with NIPPV (*p* = 0.014). *Conclusion*: LISA and INSURE are equally effective modalities for surfactant administration in neonates with no significant differences.

**Table:** 

**What Is Known:** *• Less invasive surfactant administration (LISA) is currently recommended by the latest European guidelines as the preferred method for surfactant delivery in spontaneously breathing infants on non-invasive ventilation (NIV).* *• Previous studies suggested that LISA and INSURE have similar safety and effectiveness, with comparable short- and long-term outcomes.*	
**What is New:** *• In this study, the need for a second dose of surfactant was explicitly considered as a marker of LISA failure.* *• LISA and INSURE techniques are comparable in reducing the need for mechanical ventilation and have similar major clinical outcomes, including BPD. There was a trend toward a higher rate of surfactant retreatment in the LISA group, without reaching statistical significance (p = 0.095). This was not associated with increased intubation or adverse outcomes, suggesting a potential stepwise non-invasive management strategy rather than true therapeutic failure.*

**Supplementary Information:**

The online version contains supplementary material available at 10.1007/s00431-026-07118-8.

## Introduction

Respiratory distress syndrome (RDS) is the most common respiratory disease in neonates admitted to Neonatal Intensive Care Units (NICUs), affecting primarily preterm newborns and occasionally term infants. RDS is due to deficiency of surfactant (either inadequate surfactant production or surfactant inactivation), and its incidence is inversely related to both gestational age (GA) and birth weight (BW) [1]. European Consensus Guidelines on the Management of Respiratory Distress Syndrome suggest non-invasive respiratory support (NRS) as first-line respiratory support in all babies at risk of RDS [2]. Among non-invasive ventilation (NIV) modes, nasal continuous positive airway pressure (nCPAP) and nasal intermittent positive pressure ventilation (NIPPV) are recommended as methods of choice and high-flow nasal cannulae (HFNC) as a valid alternative [3]. Surfactant replacement therapy (SRT) plays an essential role in the management of RDS as well as of other respiratory conditions (e.g. pneumonia, meconium aspiration syndrome - MAS, pulmonary haemorrhage). Prophylactic surfactant is administered shortly after birth to prevent RDS in high-risk infants (especially < 24 weeks of gestation [2]), while rescue (or selective) surfactant is given to neonates who have already developed RDS. The latter can be distinguished into early rescue (within the first 2 h of life) and delayed rescue. Early rescue showed a decreased risk of mortality and acute and chronic lung disease [4–6]. A higher starting dose (200 mg/kg) of Poractant alfa (natural surfactant) is recommended, followed, if clinical signs of respiratory distress persist, by a maximum of two other doses of lower dosage (100 mg/kg). Exogenous surfactant may be administered via different techniques: INtubate, SURfactant, Extubate (INSURE); less invasive surfactant administration (LISA); minimally invasive surfactant therapy (MIST); surfactant administration through laryngeal or supraglottic airway (SALSA); pharyngeal instillation; nebulization. LISA and INSURE are currently the most widely used techniques, showing similar safety and effectiveness [7, 8]. INSURE consists in surfactant administration after endotracheal intubation, followed by hand-bag or mechanical ventilation (MV) with positive pressure and rapid extubation. It is a well-established technique, also in centres with limited expertise, and ensures better airway control. However, it requires sedation and intubation with a potential risk of extubation failure and procedure-related complications such as hemodynamic instability, bradycardia, and increased intracranial pressure. LISA uses a thin catheter inserted into the trachea via video-laryngoscopy in spontaneously breathing neonates on NIV, without intubation and sedation. Nonetheless, LISA requires specific training and video-laryngoscopic guidance. Variability in catheter type, delivery method, and premedication protocols contributes to heterogeneous success rates. LISA failure requiring transition to INSURE or invasive ventilation may occur, especially among extremely preterm infants [9]. Although many neonatologists choose and encourage INSURE technique, the latest European guidelines recommend using a thin catheter as the preferred method of surfactant administration for spontaneously breathing babies on nCPAP [2]. Indeed, several studies [10–16] showed that less invasive modes of early surfactant administration combined with NIV decrease the need for MV within the first 72 h of life and lower the incidence of bronchopulmonary dysplasia (BPD) at 36 weeks of GA, death, and short- and long-term morbidities in high-risk preterm infants. The aim of this study was to compare the effectiveness of LISA and INSURE for rescue surfactant administration in spontaneously breathing neonates on NIV.

## Materials and methods

### Study design

This was a multicentre retrospective registry-based cohort study. The STrengthening the Reporting of OBservational studies in Epidemiology (STROBE) guidelines for reporting of observational studies were followed [17].

### Setting

The study was performed in three Southern Italy NICUs: University Hospital Policlinico (Bari), Di Venere Hospital (Bari) and Ecclesiastical General Hospital “F. Miulli” (Acquaviva delle Fonti). Infants born between January 2024 and March 2025 were enrolled. Clinical management followed local protocols in each participating centre.

### Participants

Infants of any GA were eligible if they received NIV soon after birth and rescue surfactant via either INSURE or LISA. The exclusion criteria were intubation and MV at birth and major congenital anomalies.

### Data sources

Data were gathered from medical notes and the Neocare software (GPI SpA, Trento, Italy) and entered into a Microsoft Excel file. Parental written informed consent was obtained for all patients. All data sets were anonymized.

### Study size

No formal sample size calculation was performed due to the retrospective nature of the study. The sample size was determined by the number of eligible patients during the study period.

### Variables

Neonates showing clinical and radiographic features of respiratory distress soon after birth were treated with HFNC, nCPAP or NIPPV, according to the protocols of each centre. Early targeted surfactant (200 mg/kg of Poractant alpha; Chiesi Farmaceutici, Parma, Italy) was administered to infants with worsening respiratory distress, as indicated by non-invasive respiratory support with a mean airway pressure (MAP) of ≥ 6 cm H_2_O and FiO_2_ of ≥ 0.3 to reach SaO_2_ targets. The technique for surfactant administration was decided by the neonatologist on duty. Infants receiving INSURE were orally intubated, and surfactant was instilled in the endotracheal tube in 30 to 60 s, then, after a short time of positive pressure ventilation (PPV) with hand-bag or mechanical ventilation, they were rapidly extubated onto NIV. Procedural sedation with intranasal or intravenous midazolam, intravenous fentanyl or intranasal dexmedetomidine was administered according to the clinician’s decision. LISA was performed inserting SurfCath catheter (Vygon®, Paris, France) into the trachea via video-laryngoscopy, with no need of Magill forceps and/or any sedation, in spontaneously breathing neonates on NIV. Surfactant was instilled into the catheter in roughly 60 s [18], followed by removal of SurfCath. During the procedure, NIV was not discontinued. Both procedures were performed by either a trained neonatologist or a senior resident under direct consultant supervision. Intravenous or oral caffeine citrate (20 mg/kg loading dose and 5–10 mg/kg/day as maintenance dose) was routinely started in all neonates < 32 weeks of GA for prevention of apnoea of prematurity.

For each newborn, both maternal and neonatal demographic and anamnestic data regarding pregnancy, delivery, and the postnatal period were collected, including neonatal comorbidities (i.e. infectious, haematological and cardiological disorders, intrauterine growth restriction - IUGR, gestational diabetes, and twin-to-twin transfusion syndrome - TTTS). Illness severity was assessed using the Score for Neonatal Acute Physiology with Perinatal Extension-II (SNAPPE-II), calculated within the first 12 h of life.

The primary outcome of our study was to compare LISA and INSURE in terms of need for a second dose of surfactant (100 mg/kg of Poractant alpha; Chiesi Farmaceutici, Parma, Italy) or intubation and MV within 72 h of life. Surfactant retreatment was considered in case of worsening respiratory status, particularly in the presence of increasing oxygen requirement (FiO₂ ≥ 0.30) and inadequate oxygen saturation. The surfactant administration technique was at the discretion of the attending neonatologist. Intubation was performed in case of clinical deterioration, including persistent oxygen desaturation (SpO₂ < 90%) despite FiO₂ > 0.40, respiratory acidosis (i.e. pH < 7.20, pCO_2_ > 60 mmHg), marked signs of respiratory distress or prolonged/repeated apnoeas.

The secondary outcomes were as follows: the incidence of BPD at 36 weeks post-menstrual age or at discharge, according to the National Institute of Health (NIH) 2001 definition [19] and the recent update of Jensen et al. [20]; the comparison of the three NIV modes (HFNC, nCPAP, and NIPPV) in neonates receiving LISA in terms of need for a second dose of surfactant, intubation rate within 72 h of life and incidence of BPD at 36 weeks post-menstrual age or at discharge.

Additionally, we collected data regarding other adverse outcomes, i.e. the need for intubation at any point, the duration of NIV and oxygen supplementation, the duration of MV, the need for high-frequency oscillatory ventilation (HFOV) and/or nitric oxide (NO), the incidence of air leaks (pneumothorax - PNX, pneumomediastinum, or pulmonary interstitial emphysema - PIE), treatment with postnatal corticosteroids, and death.

### Bias

Potential sources of bias include selection bias related to the clinician’s choice of surfactant administration technique, as well as variability in clinical management and respiratory support strategies across centres.

The retrospective design and the small sample size limit the statistical power of the study. The lack of standardized protocols for sedation and NIV application may have also influenced the outcomes.

### Statistical analysis

Data are reported as mean ± standard deviation (SD), median, and interquartile range (IQR). Normality was assessed by Shapiro–Wilk test. Categorical data were described as frequencies and percentage. Characteristics of the LISA and INSURE groups were compared with Student’s *t*-test or Mann–Whitney test (continuous variables) and chi-squared or Fisher exact test (categorical data) as appropriate. The analyses were carried out using STATA software, version 16 (StataCorp, College Station, TX, USA). A *p*-value of < 0.05 was considered statistically significant.

## Results

### Enrolment and demographics

**Fig. 1:**
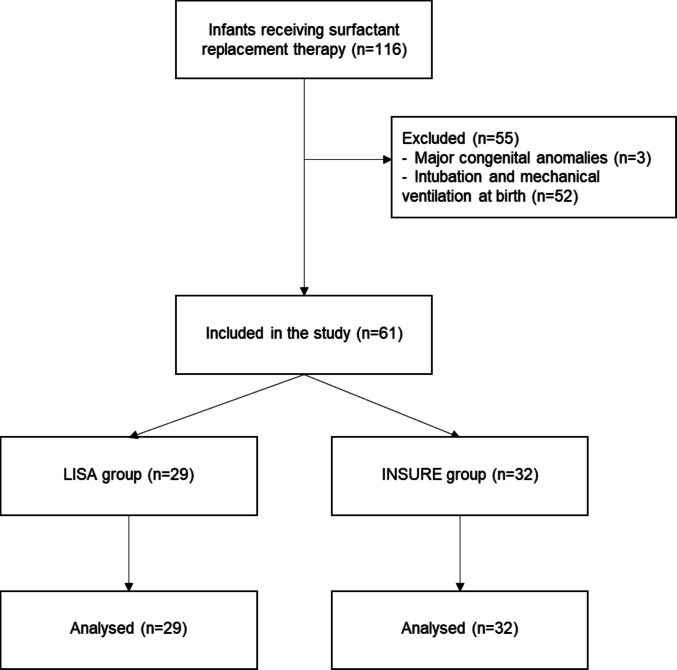
STROBE flow diagram

Between January 2024 and March 2025, 56 newborns admitted to University Hospital Policlinico NICU received SRT; 25 infants were excluded: 1 because of major congenital anomalies and 24 because of intubation at birth. At Di Venere Hospital NICU, 43 neonates received SRT and 19 were excluded: 1 because of major congenital anomalies and 18 because of intubation at birth. At “Miulli” Hospital NICU, 17 infants received SRT and 11 were excluded: 1 because of major congenital abnormalities and 10 because of intubation at birth (Fig. 1). A total of 61 newborns, 31 from University Hospital Policlinico, 24 from Di Venere Hospital, and 6 from “Miulli” Hospital, were included in the analysis. Of those, 29 neonates received surfactant via LISA method and 32 neonates via INSURE technique. No immediate adverse events linked to surfactant administration (e.g. apnoea, bradycardia, hypotension, urgent need to redirect towards intubation) were reported with either technique.

Demographic characteristics of the infants in the LISA group and in the INSURE group are shown in Table 1. There were no significant differences between the two groups regarding gender, neonatal comorbidities, rate of caesarean section, incidence of premature rupture of membranes (PROM) > 24 h, clinical diagnosis of chorioamnionitis, multiple birth, 5′ Apgar score, and administration of caffeine citrate within the first 24 h of life. Baseline illness severity, assessed by SNAPPE-II score, was comparable between groups (LISA group median 8, IQR 5–15 vs INSURE group median 7, IQR 5–12; *p* = 0.874). Mean BW and mean GA did not significantly differ between the LISA group and the INSURE group (1585 ± 607 g vs 1796 ± 687 g,* p* = 0.210, and 31.5 ± 3.6 vs 31.8 ± 3.0 weeks, *p* = 0.683, respectively). The proportion of neonates born < 32 weeks’ GA was also comparable: 59% in the LISA group vs 53% in the INSURE group (*p* = 0.797). Importantly, underlying conditions potentially contributing to respiratory distress affected 8/27 neonates born > 32 weeks’ GA (30%), including gestational diabetes (*n* = 5, of whom 2 were associated with pneumonia), TTTS (*n* = 2; 1 donor twin and 1 recipient twin), and MAS (*n* = 1). Moreover, 2/34 infants (6%) born < 32 weeks’ GA presented underlying conditions (1 gestational diabetes and 1 TTTS donor twin). The distribution of small for gestational age (SGA) and large for gestational age (LGA) neonates did not differ between groups. Infants with complete antenatal steroid prophylaxis were higher in the LISA group compared to the INSURE group (66% vs 34%, *p* = 0.015). Similarly, the percentage of primiparous mothers was significantly higher in the LISA group than in the INSURE group (59% vs 31%, *p* = 0.032). Different modes of NIV were used in the two cohorts: HFNC, nCPAP, and NIPPV. However, the proportion of babies treated with each modality did not differ among groups (HFNC 38% vs 47%, *p* = 0.481; nCPAP 14% vs 6%, *p* = 0.411; NIPPV 48% vs 47%, *p* = 0.913). Clinical severity of respiratory distress pre-SRT, assessed as oxygen requirement, pH and pCO2, was also similar (Table 2).

**Table 1:** Neonatal and maternal demographics

	Total *n*= 61	LISA *n*= 29	INSURE *n* = 32	*p*
Comorbidities, *n* (%)	21 (35)	12 (43)	9 (28)	0.233
Caucasian ethnicity, *n* (%)	59 (97)	28 (97)	31 (97)	1.000
Primigravida, *n* (%)	27 (44)	17 (59)	10 (31)	0.032
Complete antenatal steroids, *n* (%)	30 (49)	19 (66)	11 (34)	0.015
Caesarean section, *n* (%)	57 (93)	27 (93)	30 (94)	1.000
PROM > 24 h, *n* (%)	4 (7)	0 (0)	4 (13)	0.114
Clinical chorioamnionitis, *n* (%)	7 (11)	1 (3)	6 (19)	0.106
GA weeks, mean ± SD	31.6 ± 3.3	31.5 ± 3.6	31.8 ± 3.0	0.683
BW g, mean ± SD	1696 ± 653	1585 ± 607	1796 ± 687	0.210
SGA, *n* (%)	5 (8)	4 (14)	1 (3)	0.181
LGA, *n* (%)	8 (13)	3 (10)	5 (16)	0.709
Male sex, *n* (%)	29 (48)	13 (45)	16 (50)	0.686
Multiple birth, *n* (%)	23 (38)	12 (41)	11 (34)	0.573
Apgar 5', median (IQR)	9.0 (8.0–9.0)	9.0 (8.0–9.0)	9.0 (8.0–9.0)	0.719
Caffeine within first 24 h, *n* (%)	46 (75)	24 (83)	22 (69)	0.204
SNAPPE-II score, median (IQR)	8 (5–13)	8 (5–15)	7 (5–12)	0.874

*PROM* premature rupture of membranes, *GA* gestational age, *SD* standard deviation, *BW* birth weight, *SGA* small for gestational age, *LGA* large for gestational age, *IQR* interquartile range, *SNAPPE-II* Score for Neonatal Acute Physiology with Perinatal Extension-II

**Table 2:** Respiratory support and indices of respiratory distress severity

	Total *n*= 61	LISA *n*= 29	INSURE *n* = 32	*p*
HFNC, *n* (%)	26 (43)	11 (38)	15 (47)	0.481
nCPAP, *n* (%)	6 (10)	4 (14)	2 (6)	0.411
NIPPV, *n* (%)	29 (48)	14 (48)	15 (47)	0.913
pH pre-surfactant, mean ± SD	7.271 ± 0.067	7.266 ± 0.065	7.276 ± 0.070	0.596
pCO2 pre-surfactant, median (IQR)	49 (42–56)	49 (44–53)	48 (38–59)	0.869
FiO_2_ pre-surfactant %, median (IQR)	33 (30–40)	30 (30–40)	35 (30–40)	0.244

*HFNC* high-flow nasal cannulae, *nCPAP* nasal continuous positive airway pressure, *NIPPV* nasal intermittent positive pressure ventilation, *SD* standard deviation, *IQR* interquartile range

### Main outcome

The need for a second dose of surfactant was not different (*p* = 0.095): 38% (11/29) in the LISA group and 19% (6/32) in the INSURE group. Notably, one neonate in the LISA group received the second dose after escalation to invasive mechanical ventilation (IMV). Among infants requiring surfactant retreatment, those initially treated with LISA were significantly more likely to receive the second dose using the LISA technique compared with infants initially treated with INSURE (21% vs 3%, *p* = 0.046) (Table 3). No statistically significant differences were found in the need for mechanical ventilation in the first 72 h of life between the two groups: LISA 17% (5/29) and INSURE 9% (3/32), *p* = 0.460. Data are shown in Table 3.

**Table 3:** Main outcomes

	Total *n* = 61	LISA *n*= 29	INSURE *n*= 32	*p*
Surfactant second dose, *n* (%)	17 (28)	11 (38)*	6 (19)	0.095
Second dose via INSURE, *n* (%)	9 (15)	4 (14)*	5 (16)	1.000
Second dose via LISA, *n* (%)	7 (11)	6 (21)*	1 (3)	0.046
Intubation within 72 h, *n* (%)	8 (13)	5 (17)*	3 (9)	0.460

*1/11 neonate in the LISA group received the second dose during IMV

### Secondary outcomes

There were no differences in terms of BPD, duration of oxygen supplementation, duration of invasive ventilation, rates of air leaks, need for post-natal steroids and death between groups (Table 4).

**Table 4:** Secondary and adverse outcomes

	**Total** ***n* = 61**	**LISA** ***n* = 29**	**INSURE** ***n* = 32**	***p***
Respiratory acidosis, *n* (%)	25 (45)	12 (43)	13 (48)	0.694
Apnoea, *n* (%)	1 (2)	0 (0)	1 (3)	1.000
Need for intubation, *n* (%)	8 (13)	5 (17)	3 (9)	0.460
HFOV, *n* (%)	2 (3)	2 (7)	0 (0)	0.222
NO, *n* (%)	2 (3)	1 (3)	1 (3)	1.000
BPD at 36 weeks, *n* (%)	3 (5)	2 (7)	1 (3)	0.600
Air leak, *n* (%)	2 (3)	1 (3)	1 (3)	1.000
Days of supplemental O_2_ therapy (*n*), median (IQR)	6 (3–12)	7 (4–13)	6 (3–11)	0.518
Post-natal steroids for BPD, *n* (%)	4 (7)	1 (3)	3 (9)	0.614
Days of NIV (*n*), median (IQR)	7 (5–10)	7 (5–12)	7 (5–10)	0.409
Days of IMV (*n*), median (IQR)	4 (2–6)	5 (3–8)	4 (2–4)	0.371
Death, *n* (%)	1 (2)	1 (3)	0 (0)	0.475

*HFOV* high-frequency oscillatory ventilation, *NO* nitric oxide, *BPD* bronchopulmonary dysplasia, *IQR* interquartile range, *NIV* non-invasive ventilation, *IMV* invasive mechanical ventilation

In order to evaluate the outcome according to NIV mode, newborns belonging to the LISA group were further divided in subgroups according to the NIV applied: HFNC, nCPAP, and NIPPV. Demographic data showed no statistically significant differences between the three groups, except for the oxygen requirement before surfactant replacement therapy (HFNC median 40%, IQR 30–50; nCPAP median 38%, IQR 27–53; NIPPV median 30%, IQR 28–30; *p* = 0.012). However, there were no differences in main, secondary, and adverse outcomes between the three groups, except for the shorter duration of oxygen supplementation in the NIPPV group (HFNC median 10, IQR 6–14; nCPAP median 22, IQR 10–113; NIPPV median 4, IQR 2–5; *p* = 0.014) (data not shown).

## Discussion

The primary objective of this study was to compare the effectiveness of both LISA and INSURE for spontaneously breathing neonates treated with NIV who need rescue surfactant administration. Actually, LISA represents the method of choice for surfactant delivery in the latest European guidelines [2]. Indeed, many reports suggest that LISA allows lower rates of IMV compared to INSURE [10–12, 21–23]. Our analysis showed no statistically significant differences between LISA and INSURE in terms of NIV failure (defined as the need for IMV within the first 72 h of life) (LISA 17% vs INSURE 9%, *p* = 0.460), consistent with findings of recent randomized controlled trials (RCTs) [24, 25]. Although our study has lower statistical power compared to RCTs, it reflects real-world clinical practice and offers a pragmatic insight into the implementation of these techniques in routine neonatal care.

Notably, the most significant observation in our cohort was the trend towards a higher surfactant retreatment rate (i.e. administration of additional surfactant doses) in the LISA group compared to the INSURE group (38% vs 19% respectively, *p* = 0.095). In this study, the need for a second surfactant dose was explicitly considered as a marker of LISA failure, providing a clinically relevant metric to evaluate the effectiveness of the initial dose. However, the higher retreatment rate observed with LISA may reflect a stepwise non-invasive management strategy rather than true treatment failure, as it was not associated with increased intubation or adverse respiratory outcomes. Although the need for multiple surfactant doses has been consistently associated with adverse neonatal respiratory outcomes, current evidence suggests that this association primarily reflects baseline disease severity rather than surfactant retreatment acting as an independent predictor [26, 27]. Overall, we report a 28% surfactant retreatment rate. Given the GA distribution in our cohort (56% neonates born < 32 weeks), this finding aligns with literature data [28, 29], which report a first-dose failure rate of approximately 5–10% in late preterm/term neonates and 30–40% in infants born at less than 32 weeks’ gestation. In addition, a minority of infants (10/61, 16%) presented underlying conditions potentially contributing to respiratory distress severity, including MAS, pneumonia, TTTS, and maternal gestational diabetes.

The observed difference in retreatment rates may arise from two factors: first, the less controlled drug distribution via the LISA catheter, and second, differences in patient management during the procedure. Experimental models and imaging studies have suggested that less invasive techniques may lead to a less homogeneous intrapulmonary distribution of surfactant, potentially reducing its initial effectiveness [30]. In addition, the effectiveness of LISA may be influenced by operator experience and procedural variability, as well as by the absence of sedation. Since infants in the INSURE group are regularly sedated while those in the LISA group are not, a greater control over the infant’s activity (crying, coughing) due to sedation may promote a more successful and less turbulent delivery of the surfactant during intubation, a factor possibly explaining the lower retreatment rate observed with INSURE.

It is reassuring that no significant differences were observed between the two groups regarding significant adverse secondary outcomes, including the incidence of BPD at 36 weeks or at discharge (low in both groups), the duration of oxygen supplementation and invasive ventilation, incidence of air leaks, need for post-natal steroids, and death.

Furthermore, our study showed that the type of NIV influences the clinical course: within the LISA cohort, the duration of oxygen supplementation was significantly shorter in the subgroup treated with NIPPV compared to nCPAP and HFNC (*p* = 0.014). This reinforces the importance of using more active support modalities to optimize surfactant efficacy, irrespective of the administration technique.

It is essential to evaluate these results according to some methodological limits. Our study is a retrospective analysis with possible selection bias (the clinical decision to use LISA or INSURE may have been influenced by the neonate’s initial stability).

Besides, the absence of standardized protocols of sedation during the procedure represents a significant limitation. The different type of sedation (only in the INSURE group) could have acted as a confounding factor, potentially masking a true effect or magnifying the apparent success of the INSURE group due to reduced patient movement and discomfort so improving its administration. Moreover, different modalities were used to provide the initial non-invasive respiratory support (i.e. HFNC, CPAP, NIPPV), and this might have affected the initial clinical stability of the patients.

The most significant limit is the small sample size responsible for reduced adequate statistical power to detect significant differences in outcomes with low incidence, such as NIV failure or retreatment rate. The *p*-values close to 0.05, as that found for the retreatment necessity (*p* = 0.095), suggest a possible significant effect, but a larger cohort is mandatory.

Despite the lack of statistical significance for retreatment, our study provides an important clinical implication: the increased need for a second surfactant dose in the LISA group causes increased cost of care and greater patient discomfort (due to the need for a second invasive procedure). Therefore, the choice between LISA and INSURE should be evaluated on the basis of possible benefits of LISA’s lower initial invasiveness and the potential risk of less efficient administration and the need for retreatment.

## Future research and conclusions

Based on our findings, important questions for future research arise. Specifically: could a higher dosage of surfactant administered via LISA counteract the need for a second dose? Future studies need also standardized protocols, particularly concerning the use of analgesia and sedation during both LISA and INSURE procedures, to better compare both techniques. Prospective, randomized studies with larger sample sizes are required to evaluate whether higher doses delivered through the LISA technique can determine better pulmonary surfactant distribution that can be considered the cause of the well-known single-dose efficacy of INSURE. In conclusion, our data suggest that while the LISA and INSURE techniques are comparable in reducing the need for mechanical ventilation, INSURE could be more effective in terms of lower retreatment rate. However, the higher retreatment rate observed with LISA may reflect a stepwise non-invasive management strategy rather than true treatment failure, as it was not associated with increased intubation or adverse respiratory outcomes.

## Supplementary Information

Below is the link to the electronic supplementary material.

ESM 1 (134 KB PDF)

## Data Availability

The datasets analysed during the current study are available from the corresponding author on reasonable request.

## Abbreviations

BPD: Bronchopulmonary dysplasia
BW: Birth weight
GA: Gestational age
HFOV: High-frequency oscillatory ventilation
HNFC: High-flow nasal cannulae
IMV: Invasive mechanical ventilation
INSURE: INtubate, SURfactant, Extubate
IQR: Interquartile range
IUGR: Intrauterine growth restriction
LGA: Large for gestational age
LISA: Less invasive surfactant administration
MAP: Mean airway pressure
MAS: Meconium aspiration syndrome
MIST: Minimally invasive surfactant therapy
MV: Mechanical ventilation
nCPAP: Nasal continuous positive airway pressure
NICU: Neonatal Intensive Care Unit
NIH: National Institute of Health
NIPPV: Nasal intermittent positive pressure ventilation
NIV: Non-invasive ventilation
NO: Nitric oxide
NRS: Non-invasive respiratory support
PIE: Pulmonary interstitial emphysema
PNX: Pneumothorax
PPV: Positive pressure ventilation
PROM: Premature rupture of membranes
RCT: Randomized controlled trial
RDS: Respiratory distress syndrome
SALSA: Surfactant administration through laryngeal or supraglottic airway
SD: Standard deviation
SGA: Small for gestational age
SNAPPE-II: Score for Neonatal Acute Physiology with Perinatal Extension-II
SRT: Surfactant replacement therapy
STROBE: STrengthening the Reporting of OBservational studies in Epidemiology
TTTS: Twin-to-twin transfusion syndrome
