# Supplementary material for: Comparison of LISA and INSURE techniques for surfactant administration: a multicentre retrospective study
Source: Eur J Pediatr. 2026 Jun 3;185(7):461. doi: 10.1007/s00431-026-07118-8 (PMC13230275; doi:10.1007/s00431-026-07118-8)
Supplement: Supplementary file 1 — (134 KB PDF) [file 431_2026_7118_MOESM1_ESM.pdf]

STROBE Checklist for the manuscript **"Comparison of LISA and INSURE techniques for surfactant administration: a multicentre retrospective study"**

	Item No	Location in manuscript
**Title and abstract**	1	(*a*) Title (p. 1)
		(*b*) Abstract (p. 1-2)
**Introduction**		
Background/rationale	2	Introduction (p. 4-5)
Objectives	3	Introduction (p. 5)
**Methods**		
Study design	4	Materials and Methods – Study design (p. 5)
Setting	5	Materials and Methods – Setting (p. 5)
Participants	6	Materials and Methods – Participants (p. 5)
Variables	7	Materials and Methods – Variables (p. 5-6)
Data sources/ measurement	8	Materials and Methods – Data sources (p. 5)
Bias	9	Materials and Methods – Bias (p. 7); Discussion (p. 13-15)
Study size	10	Materials and Methods – Study size (p. 5)
Quantitative variables	11	Materials and Methods – Statistical analysis (p. 7); continuous variables were analysed as mean ± SD or median (IQR) according to distribution.
Statistical methods	12	Materials and Methods – Statistical analysis (p. 7)
**Results**		
Participants	13	Results - Enrolment and demographics (p. 7-9); Figure 1 (p. 8)
Descriptive data	14	Results – Enrolment and demographics (p. 7-9); Table 1 (p. 9-10)
Outcome data	15	Results – Tables 2-4 (p. 11-13)
Main results	16	Results – Main outcome and Secondary outcomes (p. 11-13)
Other analyses	17	Results (p. 8-13)
**Discussion**		
Key results	18	Discussion (p. 13-15); Future Research and Conclusions (p. 15)
Limitations	19	Discussion (p. 13-15)
Interpretation	20	Discussion (p. 13-15)
Generalisability	21	Discussion (p. 13-15); Future Research and Conclusions (p. 15)
**Other information**		
Funding	22	Statements and Declarations - Funding (p. 19)
